# Supplementary material for: Fabrication of ultrahigh-precision hemispherical mirrors for quantum-optics applications
Source: Sci Rep. 2018 Jan 9;8:221. doi: 10.1038/s41598-017-18637-8 (PMC5760700; doi:10.1038/s41598-017-18637-8)
Supplement: Supplementary file 1 — Supplementary information [file 41598_2017_18637_MOESM1_ESM.pdf]

Fabrication of ultrahigh-precision hemispherical mirrors for quantum-optics applications: supplementary material

Daniel B. Higginbottom[1,2,*], Geoff T. Campbell[1], Gabriel Araneda[2], Fengzhou Fang[3], Yves Colombe[2], Ben C. Buchler[1], and Ping Koy Lam[1,†]

[1]Centre for Quantum Computation and Communication Technology, Research School of Physics and Engineering, The Australian National University, Canberra ACT 2601, Australia.
[2]Institut für Experimentalphysik, Universität Innsbruck, Technikerstr. 25, 6020 Innsbruck, Austria
[3]State Key Laboratory of Precision Measuring Technology & Instruments, Centre of MicroNano Manufacturing Technology, Tianjin University, Tianjin 300072, China
[*]daniel.higginbottom@anu.edu.au
[†]ping.lam@anu.edu.au

ABSTRACT

This document provides supplementary information to “Fabrication of ultrahigh-precision hemispherical mirrors for quantum-optics applications”, Scientific Reports 8, Article number: 221 (2018), https://www.nature.com/articles/s41598-017-18637-8. It contains derivations of the analytic error models used in the body of the paper, a detailed description of the interferometric calibration method used to correct for such offsets, and additional data showing the deformation of a hemispheric mirror during bake-out for an ultra-high vacuum experiment.

X-offset error profile

During the cut the tool describes a near-perfect circle with radius R_c given by the distance from the cutting edge to the center of rotation, limited by the radial and axial displacement of the lathe’s B-axis over 90° rotation (both specified as less than 50 nm over 360°). However, the actual profile cut in the X-Z plane depends on the offset Δx between the tool rotation (B) axis and part rotation (C) axis (see Fig. S2a). In Cartesian coordinates the cut profile is

$$z_c = \sqrt{R_c^2 - (|x| - \Delta x)^2}\,. \tag{1}$$

We are interested in how closely this profile matches a circle with radius R_f and CoC displaced from the B-axis center by distance Δz (the blue circle in Fig. S2a) given by

$$z_f = \sqrt{R_f^2 - x^2} + \Delta z\,. \tag{2}$$

To find the radial displacement between these two surfaces we transform the Cartesian curves z_c and z_f into radial profiles in spherical coordinates

$$r_c(\phi) = R_c\sqrt{1 - \frac{1}{2}\left(\frac{\Delta x}{R_c}\right)^2 (1 + \cos 2\phi)} + \Delta x \sin\phi\,, \tag{3}$$

$$r_f(\phi) = R_f\sqrt{1 - \frac{1}{2}\left(\frac{\Delta z}{R_f}\right)^2 (1 - \cos 2\phi)} + \Delta z \cos\phi\,. \tag{4}$$

In the limit $R_c, R_f \gg \Delta x, \Delta z$ the square-root terms approach one and the error profile, the difference between the cut and fit radial profiles, simplifies to

$$r_e(\phi) = r_f(\phi) - r_c(\phi) = \Delta R - \Delta z \cos\phi - \Delta x \sin\phi\,, \tag{5}$$

where $\Delta R = R_f - R_c$. We can integrate over the surface area of a sphere with NA $= \sin\phi_a$ to find the RMS error

$$E_{\mathrm{RMS}} = \sqrt{\int_0^{\phi_a} r_e^2 \sin\phi\, d\phi / (1 - \cos\phi_a)}\,, \tag{6}$$

where the area element is weighted by $\sin\phi$ and the integral over θ yields a normalization factor $1-\cos\phi_a$. For a complete hemisphere (NA = 1, $\phi_a = \pi/2$) this reduces to

$$E_{\mathrm{RMS}} = \sqrt{\Delta R^2 + \frac{2}{3}\Delta x^2 + \frac{1}{3}\Delta z^2 + \frac{\pi}{2}\Delta x\Delta R + \Delta z\Delta R + \frac{2}{3}\Delta x\Delta z}\,. \tag{7}$$

The best-fit spherical surface is given by the parameters ΔR_{xfit} and Δz_{xfit} that minimize the RMS error for a given Δx

$$\Delta R_{\mathrm{xfit}}(\phi_a = \pi/2) = (\pi-2)\Delta x\,, \tag{8}$$

$$\Delta z_{\mathrm{xfit}}(\phi_a = \pi/2) = \frac{1}{2}(3\pi-8)\Delta x\,, \tag{9}$$

which are plotted in Fig. 2c (red and green lines) as a function of Δx. With these optimal parameters the radial error profile is

$$r_e = -\Delta x[(\frac{3\pi}{2}-4)\cos\phi + \sin\phi + (2-\pi)]\,. \tag{10}$$

This whole-hemisphere X-calibration error profile is plotted in Fig. 2a, and used to infer the residual offset of the finished hemispheres from their measured surface profile. In this way we are able to infer the accuracy of our calibration technique (see the main text). We can calculate the RMS error and PV error from this profile (Fig. 2c, black and blue lines) to give the X-calibration error budget rule of thumb used in the text

$$\begin{aligned} E_{\mathrm{RMS}} &= |\Delta x|\sqrt{\frac{3\pi(\pi-4)-8}{12}} \approx \frac{|\Delta x|}{11}\,, \\ E_{\mathrm{PV}} &= \frac{|\Delta x|}{2}(8-3\pi+\sqrt{68-48\pi+9\pi^2}) \approx \frac{|\Delta x|}{2}\,. \end{aligned} \tag{11}$$

The above expressions are true for a complete hemisphere (NA $=1$), but in general the best-fit sphere parameters ΔR_{xfit} and Δz_{xfit} depend on the numerical aperture of the surface section being measured. For a spherical surface with NA $=\sin\phi_a < 1$ the parameters ΔR and Δz that satisfy

$$\frac{\partial E_{\mathrm{RMS}}}{\partial \Delta R} = \frac{\partial E_{\mathrm{RMS}}}{\partial \Delta z} = 0 \tag{12}$$

are

$$\begin{aligned} \Delta R_{\mathrm{xfit}}(\phi_a) &= \frac{\Delta x}{4}\csc^6\left(\frac{\phi_a}{2}\right)\left(\frac{3\phi_a}{2} - \sin\phi_a + \sin 2\phi_a + \phi_a\cos\phi_a + \frac{\phi_a}{2}\cos 2\phi_a\right)\,, \\ \Delta z_{\mathrm{xfit}}(\phi_a) &= \frac{\Delta x}{32}\csc^8\left(\frac{\phi_a}{2}\right)\sin^2\phi_a\left(6\phi_a - 8\sin\phi_a + \sin 2\phi_a\right)\,, \end{aligned} \tag{13}$$

which are very closely approximated by their first order Taylor expansions in ϕ_a about $\phi_a = 0$

$$\begin{aligned} \Delta R_{\mathrm{xfit}}(\phi_a) &\approx \Delta x\left(\frac{8}{5\phi_a} + \frac{8}{105}\phi_a\right)\,, \\ \Delta z_{\mathrm{xfit}}(\phi_a) &\approx \Delta x\left(\frac{8}{5\phi_a} - \frac{4}{21}\phi_a\right)\,. \end{aligned} \tag{14}$$

By substituting these parameters into the general equation of the radial error profile, Eqn. 5, and integrating over the surface area as in Eqn. 6 we can find the aggregate errors over any measured NA as a function of the X-offset error profile. The RMS error and PV error as a function of the measured aperture are shown in Fig. 2b.

Y-offset error profile

In contrast to the X-axis, the Y-axis lies outside of the plane of B-rotation, and makes no contribution to the final form of the mirror outside of a small central defect. A Y-axis offset Δy produces a circular defect at the center of the mirror with corresponding radius Δy. This defect is either a cone or a pillar depending on the sign of Δy, and can be measured and corrected directly using the on-lathe white light interferometer. Once the defect has been identified and measured with the interferometer it can be corrected by an adjustment of the Y-axis. The small defect makes no contribution to the RMS error because it covers a negligible region of the surface.

Outside of the central defect, a Y-offset makes a small contribution to the final form by increasing the distance of the cutting edge to the center of the C (part rotation) axis

$$r_c(\phi) = \sqrt{R_c^2 + \Delta y^2}\,. \tag{15}$$

In the limit $R_c \gg \Delta y$ this reduces to

$$r_c(\phi) = R_c + \frac{\Delta y^2}{2R_c}\,, \tag{16}$$

which is a spherical surface with RoC increased by

$$\Delta R_{\text{yfit}} = \frac{\Delta y^2}{2R_c}\,. \tag{17}$$

By the same logic we see that for a combination of X and Y offsets, and for any measurement aperture, the fit parameters from Eqn. 13 become simply

$$\begin{aligned} \Delta R_{\text{fit}}(\phi_a) &= \Delta R_{\text{xfit}}(\phi_a) + \Delta R_{\text{yfit}}(\phi_a)\,, \\ \Delta z_{\text{fit}}(\phi_a) &= \Delta R_{\text{xfit}}(\phi_a) \end{aligned} \tag{18}$$

with no increase in the aggregate errors compared to an X-offset. This is illustrated in Fig. 2d where a Y-offset results in a quadratic increase in the RoC but does not contribute to the aggregate error.

X-offset calibration example

The offset Δx between the B and C axes centers is removed by an interferometric calibration process summarized in the text of the paper using the on-lathe white light interferometer shown in Fig. S1. Two 1 μm deep grooves are cut on the outer surface of the aluminum substrate with the tool at coordinates X_1, B_1 and X_2, B_2 shown in Fig. S2(a). The spindle rotation direction is reversed between the two positions. With coordinates $X_2 = -X_1$ and $B_2 = 180^\circ - B_1$ the difference in depth between the two grooves is equal to $2\Delta x$. By measuring the depth difference we determine and correct the offset.

The depth of the grooves is measured with an in-situ white-light interferometer. The interferometer is fixed stably to the tool post in order to take advantage of the lathe axes' precise positioning control. Central-fringe identification provides a relative position measurement between the interferometer objective and the mirror surface. The X-axis displacement required to shift the central fringe from the bottom of one groove to the other is the depth difference between the two grooves. After shifting the center position to $X_0' = X_0 + \Delta x$ the depth of two subsequent calibration cuts is equal. Fig. S2(b) shows an interferogram of two equal-depth calibration cuts made following an calibration step, the central maxima is simultaneously aligned with the bottom of both grooves.

Although the central-fringe maxima identifies the mirror surface position with sub-nm precision, and the lathe can be positioned with nm control precision, we infer from repeatability tests an alignment precision of 10 nm. This is consistent with the radial displacement error of the B-axis under rotation, which is specified as less than 50 nm. Furthermore, we expect additional uncertainty due to the difficulty of cutting under identical conditions at B_1 and B_2, from which we infer that the real radial uncertainty of B-rotation is less than 10 nm. This is the PV lower limit of hemispheres cut using this technique.

Bake-out test for UHV experiments

Part C was measured before and after a bake-out for UHV to determine the degree of deformation during this process. As the temperature is increased and decreased strains within the material substrate relax and produce volumetric changes that deform the spherical surface. These strains may be due to the temper of the aluminum, or may be produced by the manufacturing process. To reduce this effect the mirror substrates are temperature cycled before the surface is turned on the nano-lathe, essentially undoing the material temper. All the parts presented here were cycled from room temperature to 300° for one hour, three times before finishing.

The bake-out test was performed by heating part C to 200° celsius in vacuum for two hours. Tab. I and Fig. S3 compare the before and after measurements of the part. To produce Fig. S3 the after image is translated, rotated, and translated again until it has the maximum possible overlap with the before image. The residual difference is taken to be the distortion of the part. The final image is smoothed with a Gaussian filter (width 10 pixels) to remove artefacts of the fitting process.

The bake-out induced deformation has distorted the surface by up to 60 nm, and the RMS error is increased from 18.3 to 27.4 nm, an increase of 50%. This is a considerable distortion, although an order of magnitude smaller than the measured deformation of mirrors which were not temperature cycled before cutting.

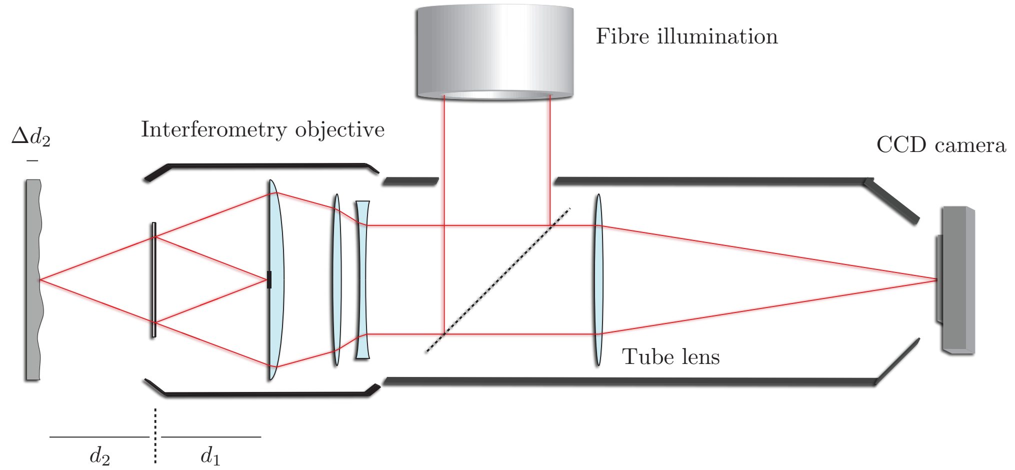

Figure S1. Schematic of the white-light interferometer used for on-lathe calibration of the lathe before diamond turning hemispheres. The objective used was a 20X Nikon CF IC Epi Plan DI interferometry objective. This was then mounted on an Infinitube FM-200 microscope body with an in-line fibre illuminator port. The white light source was a three-LED illuminator with emission at 465 nm, 521 nm and 635 nm. The distance between the objective and the work-piece was adjusted using the lathe's motion control with nanometre accuracy.

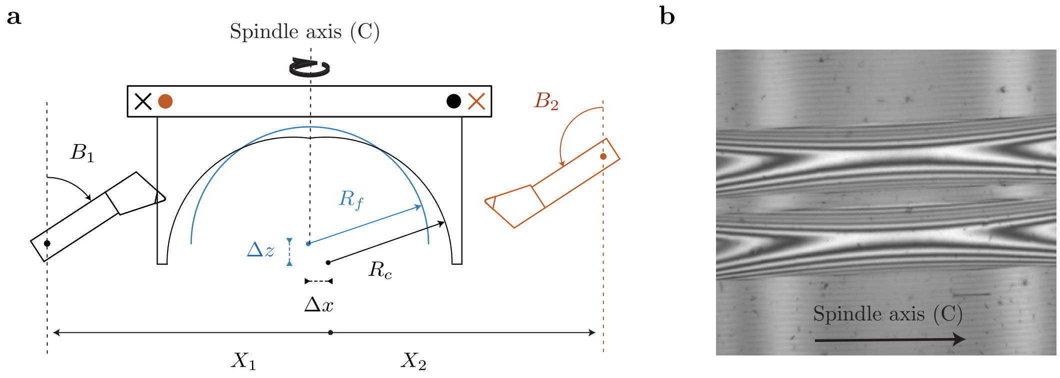

Figure S2. (a) Schematic of an (exaggerated) X-offset error profile and the corresponding calibration procedure as described in the text. The B-center is offset from the C-center by Δx and produces an aspheric surface (black). The spherical fit to this surface (blue) has RoC $R_f = R_c + \Delta R$ and CoC offset by Δz. To correct this X-offset we cut two grooves with the tool at positions $X_{1,2}, B_{1,2}$ where $X_2 = -X_1$ and $B_2 = 180^\circ - B_1$. (b) A white-light interferogram showing two calibration grooves of equal depth.

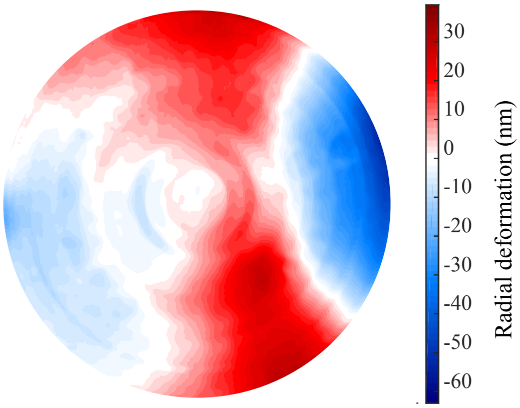

Figure S3. Deformation of part C during bake-out as an azimuthal equidistant projection.
